# Increased Circulating Chemerin in Relation to Chronic Microvascular Complications in Patients with Type 2 Diabetes

**DOI:** 10.1155/2019/8693516

**Published:** 2019-07-16

**Authors:** Ping Gu, Wei Wang, Yue Yao, Yixin Xu, Liping Wang, Pu Zang, Jian Ma, Cuihua Yang, Junya Liang, Bin Lu, Jiaqing Shao

**Affiliations:** ^1^Department of Endocrinology, Jinling Hospital, School of Medicine, Nanjing University, Nanjing, Jiangsu Province, China; ^2^Hypertension Research Center, Jiangsu Province Hospital of Chinese Medicine, Affiliated Hospital of Nanjing University of Chinese Medicine, China

## Abstract

**Objective:**

Type 2 diabetes (T2DM) is a global epidemic and increases mortality due to its vascular complications. Chemerin has been found to exert a major role in glucose and lipid metabolism. The aim of this study was to explore the correlation between plasma chemerin levels and microangiopathy in patients with T2DM.

**Methods:**

A total of 598 T2DM patients were classified into two groups: with and without microvascular complications. Anthropometric parameters and blood pressure were taken. The amounts of glycosylated hemoglobin, glucose, lipid profiles, creatinine, and chemerin concentrations in the blood were determined. The presence and severity of nephropathy, retinopathy, and neuropathy were also evaluated by specific tests.

**Results:**

Plasma levels of chemerin in diabetic subjects with microvascular complications were markedly elevated compared to those without. The number of microvascular complications increased with high plasma chemerin levels. Patients with high chemerin levels had an increased incidence of nephropathy and retinopathy. Furthermore, the chemerin plasma concentrations increased with the progression of diabetic nephropathy with highest values in macroalbuminuria groups. In contrast, no significant difference was observed in plasma chemerin levels between subjects with and without peripheral neuropathy. Pearson correlation analysis showed that plasma chemerin levels were positively related to duration of diabetes, serum creatinine, and 24-hour urine albumin excretion, even after multiple adjustments. Using logistic regression analysis, plasma chemerin concentrations were independently associated with the presence of nephropathy and retinopathy, not neuropathy.

**Conclusion:**

This study elucidated a positive correlation between increased chemerin levels and the development of some subtypes of diabetic microangiopathy.

## 1. Introduction

In recent years, the prevalence of diabetes in developing countries has increased rapidly [1]. In China, there were 114.4 million cases of diabetes reported in 2017 and the number was predicted to increase to 150 million by 2040 [2]. Diabetic micro- and macrovascular complications are major contributors to morbidity and mortality [3, 4]. Furthermore, there is evidence that micro- and macrovascular complications also occur at the stage of prediabetes [5]. Therefore, identification of biomarkers or earlier prognostic factor for vascular complications is urgently needed to prevent these complications.

Adipokines are cytokines secreted by adipose tissue and play a key role in whole glucose/lipid homeostasis. In particular, adipokines have been demonstrated to exert biological activities in vascular tissues and have an important impact on the development of vascular complications [6–9]. Chemerin is a recently discovered adipokine and has been considered a critical regulator of many biological processes, including adipocyte formation, glucose homeostasis, immune function, and inflammation [10, 11]. In humans, circulating chemerin concentration is positively related to body mass index (BMI), blood pressure (BP), triglyceride levels, fasting blood glucose, insulin resistance, and cholesterol levels [10–13], suggesting that it has a critical function in obesity and related comorbidities.

There is growing evidence indicating the association of chemerin with macrovascular diseases. A positive correlation was observed between circulating chemerin concentrations and severity of coronary atherosclerosis in patients with cardiovascular diseases [14]. It has been shown that chemerin secretion in pericoronary and periaortic adipose tissue was highly associated with atherosclerosis in their respective vessels [15]. The plasma chemerin increased significantly in patients with acute ischemic stroke and was also closely related to carotid plaque instability, suggesting that chemerin may be a potential marker for stroke [16]. Moreover, it has been reported in several cross-sectional studies that chemerin concentrations were independently correlated to subclinical atherosclerosis markers including Pulse Wave Velocity (PWV), carotid Intima Media Thickness (cIMT), and Flow-mediated Dilatation (FMD) in metabolic syndrome patients [12, 17]. These findings highlight the importance of chemerin as an independent predictor for macrovascular complications, especially atherosclerosis.

However, the role of chemerin in the development of microangiopathy is not fully understood. Microvascular complications are the major causes of kidney failure, foot ulceration and amputation, and acquired blindness due to diabetes. Some small cross-sectional studies in both Type 1 and Type 2 diabetes have found that circulating chemerin levels were increased in subjects with renal dysfunction [18, 19]. However, whether chemerin independently predicts conversion from normoalbuminuria to microalbuminuria in diabetes is not clear. So far, few studies have been published about the relationship between chemerin and retinopathy and neuropathy.

The purpose of this study is to assess whether plasma levels of chemerin are correlated with diabetic retinopathy, nephropathy, and neuropathy and to identify the potential role of chemerin as a novel risk factor for microvascular complications in an Asian Chinese diabetic population.

## 2. Subjects and Methods

### 2.1. Patients' Enrollment

In this study, we recruited 598 subjects with T2DM from inpatients and outpatients of Jinling Hospital from October 2014 to February 2017. The criteria for diabetes diagnosis were according to American Diabetes Association (ADA) Guidelines. A questionnaire was used to collect data including age, sex, age of onset of diabetes, type and dose of insulin therapy, high blood pressure, medication treatment, and other chronic and serious illness.  Table 1 summarized detailed characteristics of the entire study population. We excluded patients with a history of type 1 diabetes, diabetic macrovascular complications, acute diabetic complications, serious infectious disease, malignancy, renal and hepatic diseases, or any other severe chronic illness under treatment. The research protocol was approved by the local ethical committee of Jinling Hospital and conformed to the provisions of the Declaration of Helsinki. Written informed consent forms were obtained from all participants.

### 2.2. Anthropometric Measurement

Anthropometric parameters, that is, height, body weight, blood pressure, and waist circumference (WC) were obtained for each subject using standardized techniques. The height was recorded to the nearest 0.5 cm and the weight to the nearest 0.1 kg. WC measurement was taken to the nearest 0.1 cm at the midpoint between the inferior margin of the 12th rib and the upper border of the iliac crest. The BP measurements in the right brachial artery were acquired in subjects rested for at least 15-min by a mercury tensiometer and a mean of three measurements was used. BMI (kg/m^2^) was defined as the weight divided by the square of the height.

### 2.3. Biochemical Parameters

Blood samples were obtained after at least 10 hours of fasting overnight. After centrifugation, the supernatant was aliquoted for storage at –80°C for subsequent testing. The blood glucose concentration was analyzed using a glucose oxidase method and hemoglobin A1c (HbA1c) levels were measured with high performance liquid chromatography (G8 HPLC Analyzer). The concentrations of Triglyceride (TG), Total Cholesterol (TC), High-Density Lipoprotein Cholesterol (HDL-C), Low-Density Lipoprotein Cholesterol (LDL-C), creatinine, and high-sensitivity C-reactive protein (hsCRP) were determined by a 7600 Hitachi Automatic Analyzer (Japan). Plasma chemerin concentrations were determined using an ELISA kit (R&D Systems, Minneapolis, MN 55413, USA). The intra- and interassay CV were less than 4.5% and 6.9%, respectively.

### 2.4. Assessment of Microvascular Complications

Evaluation of diabetic nephropathy (DN), or diabetic kidney disease, was based on the presence of microalbuminuria, which was measured by the immunoturbidimetric method (Beckman Coulter IMMAGE, USA). Normoalbuminuria was defined as urinary albumin excretion (UAE) <30 mg/24h, microalbuminuria as 30 to 300 mg/24h, and macroalbuminuria as > 300 mg/24h.

Diabetic retinopathy (DR) evaluation was performed by an experienced ophthalmologist according to the American Academy of Ophthalmology definitions. DR diagnostic assessment was based on dilated ophthalmoscopy. Fluorescein fundus angiography was performed if clinically indicated. Retinopathy was classified as nondiabetic retinopathy (NDR), nonproliferative diabetic retinopathy (NPDR: intraretinal hemorrhage, microaneurysms, hard or soft exudates, and venous beading), and proliferative retinopathy (PDR: neovascularization on optic disc and/or elsewhere on the retina).

Diabetic peripheral neuropathy (DPN) was confirmed using the American Diabetes Association diagnostic criteria. Patients were asked for the symptoms or signs of motor, sensory, and autonomic nerves. A standard neurological examination was performed including the foot sensation test using monofilament or vibration and an assessment test of the ankle reflex. Nerve conduction velocity (NCV) tests were conducted by experienced clinicians on an electromyography machine (Alpine BioMed ApS, Denmark) if needed. Patients were diagnosed with peripheral neuropathy when at least two of the following symptoms presented: being insensible to 10-g Semmes-Weinstein monofilament at any of plantar sites on each foot, reduced vibration perception, absence of ankle reflex, or at least two abnormal NCV.

### 2.5. Statistical Analysis

Date analysis was performed using SPSS software version 17.0 (Chicago, IL). Continuous variables were summarized as mean ± SD or as median (first and third quartiles) if the distributions were skewed. Log transformations of hs-CRP, TG, and HDL-C were used to normalize distributions before analysis. A comparison between two or more sets of patients was analyzed with an independent t test or one-way ANOVA. Different proportions between groups were analyzed using the Chi-Square test. A Pearson correlation was applied to describe the correlation between plasma chemerin and other variables. Line and logistic regression models were used to evaluate the strength and independency of associations. A* P* value <0.05 was considered to be statistically significant.

## 3. Results

### 3.1. Baseline Characteristics of the Studied Population

The sample population was composed of 383 men and 215 women and had a mean age of 54.56 ± 12.14 years, a mean diabetes duration of 8.80±6.48 years, and an average HbA1c of 8.71±1.87% (Table 1). Among the 598 diabetic patients studied, nephropathy was present in 43.6% (microalbuminuria: 31.1%, macroalbuminuria: 12.5%), retinopathy in 34.9% (NPDR: 25.9%, PDR: 9.0%), and peripheral neuropathy in 33.6% of the subjects. Four hundred and sixty-seven patients had at least one microangiopathic complication. Of this group, three hundred and twenty-two cases had just one complication, 86 cases had two complications, and 59 cases had all three diabetic complications. As shown in Table 1, patients having one or more microvascular complications were shown to have a longer diabetes duration, poorer glycemic control, and higher Scr, hs-CRP, and chemerin levels. Otherwise, no significant differences in age, sex, BMI, WC, BP, lipid profiles, and medication were observed between patients with and without complications.

**Figure 1:**
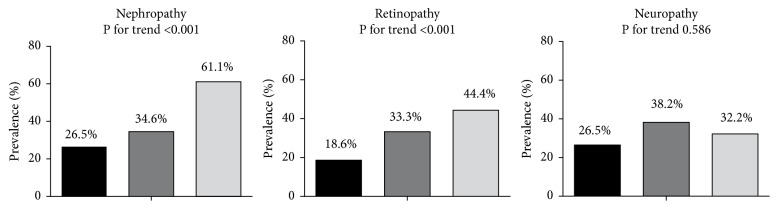
*The proportion of patients with microvascular complications across tertiles of chemerin*. As shown, significant differences across tertiles of plasma chemerin were seen for nephropathy and retinopathy, whereas no changes were seen for neuropathy. P for trend among all three tertiles is presented. P-value for significant difference between the groups was determined by the one-way analysis of variance (ANOVA).

**Figure 2:**
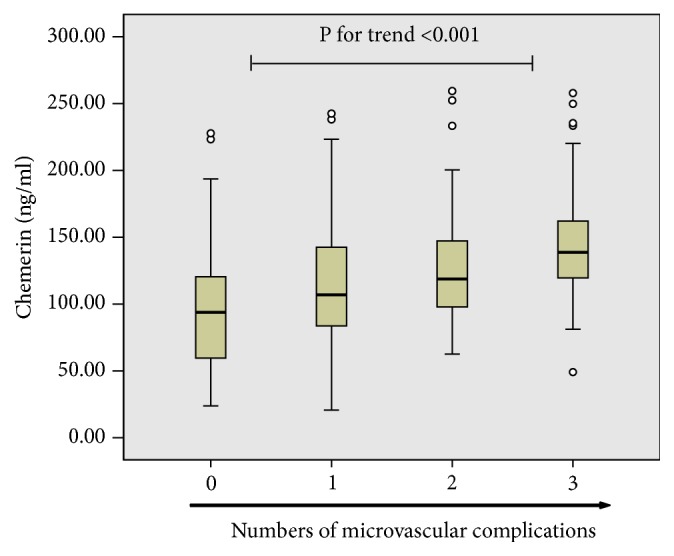
*Plasma chemerin concentrations in relation to the number of diabetic microvascular complications*. As shown, plasma chemerin concentrations elevated significantly with increasing number of microangiopathic complications in diabetes. P-value for significant difference between the groups was determined by the one-way analysis of variance (ANOVA).

The clinical and biochemical characteristics by the tertiles of chemerin levels were shown in Table 2. Patients with increased concentrations of chemerin had a longer duration of diabetes and higher BMI, Scr, 24-hour urine microalbumin, and hs-CRP levels whereas HbA1c and lipid profiles were similar among all groups. Furthermore, they exhibited an increased prevalence of retinopathy and nephropathy, but not neuropathy (Figure 1). As microvascular complications often coexist, we analyzed the association between the total number of microvascular complications (0, 1, 2, or 3) and the levels of plasma chemerin. After adjustments, plasma chemerin levels were shown to elevate significantly with the increasing number of microangiographic diabetic complications (no complications: 97.69±40.27 ng/ml, one complication: 112.29±42.69 ng/ml, two complications: 127.52±39.24 ng/ml, and three complications: 147.47±39.87 ng/mL, p<0.05, Figure 2). To investigate the association between chemerin levels and metabolic factors, a Pearson correlation was performed on all subjects. As shown in Table 3, a strong positive relationship was found between chemerin and BMI, SBP, TC, LDL-C, hs-CRP, Scr, HbA1c, 24-hour urine microalbumin, and the duration of diabetes, whereas there was no significant association with age, sex, waist circumference, DBP, and HDL-C. Then a multiple line regression analysis with all of the significant variables from Pearson's correlation showed that only BMI, SBP, Scr, hs-CRP, and 24-hour urine microalbumin remained significantly correlated with plasma chemerin concentrations.

### 3.2. Chemerin and Diabetic Nephropathy

**Figure 3:**
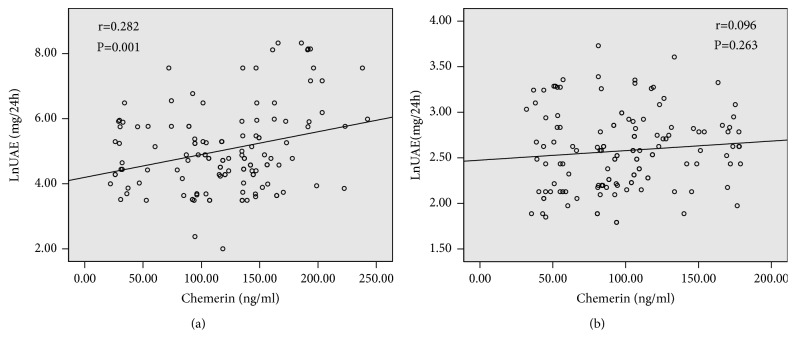
*Scatter plot showing the correlation between plasma chemerin levels and urinary albumin excretion (UAE) in patients with (a) and without nephropathy (b)*. Scatter plot with regression line showing the positive relationship between chemerin levels and UAE only in patient with nephropathy, not patient without nephropathy. Solid lines indicate regression lines. Pearson correlation was calculated in the correlation analyses.

To clarify the role of chemerin in the development of DN, we compared the data according to the severity of nephropathy. As shown in Table 4, age, sex, duration of diabetes, BMI, BP, HDL-C, LDL-C, and insulin treatment were comparable among the three groups. Plasma levels of chemerin were significantly different between the nephropathy stages and appeared to increase with the progress of diabetic nephropathy (Normoalbuminuria: 97.69±40.27ng/ml, Microalbuminuria: 112.93 ± 43.40ng/ml, and Macroalbuminuria: 135.08±65.42 ng/ml, p < 0.01). As shown in Figure 3, significant positive correlations between UAE and plasma chemerin concentrations were observed in subjects with proteinuria (r=0.282, P <0.01, Figure 3(a)), but not in nonproteinuric individuals (r=0.096, P =0.263, Figure 3(b)). Moreover, in multiple line regression analysis with UAE as the dependent variable, only duration of diabetes, HbA1c, hs-CRP, serum creatinine, and chemerin were independently associated with UAE. A trend chi-square was adopted to assess the correlation between chemerin levels and the severity of diabetic nephropathy. As a result, the plasma chemerin levels were related to the severity of nephropathy (x^2^=21.18, P<0.001). A logistic regression model revealed that elevated chemerin concentration was independently linked to the risk of developing nephropathy even after adjusting for other recognized risk factors (OR:1.802, 95% CI:1.019-3.188, p<0.05).

### 3.3. Chemerin and Diabetic Retinopathy

The result shown in Table 5 summarized the clinical parameters of the three subgroups, stratified according to diabetic retinopathy traits. There is no significant difference among the three groups in terms of age, sex, BMI, and blood pressure. We next observed the relationship between plasma chemerin concentrations and diabetic retinopathy. Plasma chemerin concentrations in patients with PDR (119.95±48.62 ng/ml) or NPDR (112.66±38.34 ng/ml) were significantly elevated as compared to patients without DR (97.69±40.27 ng/ml). However, there is no significant difference between patients with PDR or NRDR. Trend chi-square analysis also showed that there is no correlation between the plasma chemerin levels and severity of retinopathy (x^2^=6.78, P=0.148). Logistic regression analysis revealed that plasma chemerin concentration (OR:1.943, 95%CI:1.017-3.711, p<0.05), duration of diabetes (OR:1.626, 95% CI:1.28-2.06, p<0.01), total cholesterol (OR:2.002, 95% CI:1.069-3.757, p<0.05), and HbA1c (OR:1.342, 95% CI:1.211-1.606, p<0.01) were independent risk factors of retinopathy.

### 3.4. Chemerin and Diabetic Peripheral Neuropathy

The comparisons of the demographics between diabetic subjects with versus without peripheral neuropathy are shown in Table 6. The age, gender, body mass index, blood pressure, lipid profiles, and serum creatinine levels did not differ in both subgroups. Mean duration of diabetes and HbA1c were significantly higher in subjects with peripheral neuropathy than in those without. While no significant differences in levels of plasma chemerin were found between the two groups, logistic regression analysis revealed that only duration of diabetes (OR:1.81, 95% CI:1.392-2.330, p<0.01) and HbA1c (OR:1.252, 95% CI:1.036-1.519, p<0.05) were independent predictors of peripheral neuropathy.

## 4. Discussion

The major finding in our study was that T2DM with microvascular complications has higher plasma chemerin levels than the age- and sex-matched controls. Furthermore, plasma chemerin was associated with progression from none or microalbuminuria to overt proteinuria and incidence of retinopathy. And these associations were independent of known risk factors for microvascular complication, that is, duration of diabetes, glycemic control, dyslipidemia, and hypertension. However, no association of chemerin and peripheral neuropathy was found in our study. These findings indicate that plasma chemerin might serve as a significant risk factor for retinopathy and nephropathy in T2DM.

Chemerin was initially identified as a chemoattractant protein, inducing chemotaxis of various immune cells, thus playing an important role in immunity and inflammatory response [20]. Recently, it was recognized as an adipokine that was expressed in mouse and human adipocytes and was associated with obesity and metabolic syndrome [10]. Concentrations of circulating chemerin are elevated in T2DM, obesity, hypertension, dyslipidemia, and fatty liver disease [11]. Subsequent studies have also shown the pivotal role of chemerin in the pathophysiology of atherosclerosis and cardiovascular diseases [12, 14–17]. However, few studies in the literature have addressed the association of chemerin and diabetic microangiopathy.

Our previous studies have demonstrated that circulating chemerin concentrations were significantly increased in patients with T2DM. In this study, we observed that plasma level of chemerin in diabetic subjects with microvascular complications was markedly higher than in those without. Furthermore, plasma chemerin concentrations were significantly elevated with increasing number of diabetic microangiographies. And patients with high chemerin levels had more nephropathy and retinopathy, but not neuropathy. This investigation suggested that high chemerin levels are relevant to microvascular diabetic complications. To our best knowledge, this is the first report to investigate the relationship between chemerin levels and three microvascular complications simultaneously in Asian Chinese subjects with T2DM.

Recently, circulating chemerin concentrations were reported to be independently related to renal function in chronic hemodialysis patients [18]. To examine the effects of chemerin on nephropathy in diabetes, an analysis of subgroups was performed to compare their levels in different stages of diabetic nephropathy. We found that the plasma chemerin concentrations were significantly increased in subjects with macro- or microalbuminuria, compared to those with normoalbuminuria. Moreover, patients with macroalbuminuria had higher chemerin levels than those with microalbuminuria. Furthermore, the plasma chemerin level was independently related to UAE even after being adjusted for the clinical and demographic characteristics. Consistent with our study, Elsebai et al. found that circulating chemerin levels were increased significantly in diabetic patients with nephropathy [21]. These findings suggested that chemerin levels were independently related to the progression of albuminuria and renal function in diabetes. Several mechanisms underlying nephropathy have been proposed: chemerin and its receptor (ChemR23) are expressed in renal tissue [22, 23] and increased in kidney tissue of diabetic animals; binding of ChemR23, chemerin attracts immune cells to the sites of inflammation via NF-kB and MAPK pathways, stimulating their adhesion [24]; increased chemerin promotes alteration in endothelial activation and junctions correlated with CD146, possibly leading to leakage of albumin in the urine [25]; chemerin also induces secretion of inflammatory and fibrosis factors including INF-*γ*,TNF-*α*, and TGF-*β*1, facilitating kidney fibrosis and sclerosis [24, 26, 27]. The precise role of chemerin in diabetic nephropathy still needs to be elucidated by clinical and experimental studies in the future.

Previous studies demonstrated that low level chronic inflammation was a contributing factor for retinal vascular lesions in diabetes [28, 29]. Chemerin, as a possible marker of vascular inflammation, has been found to be increased in vitreous of patients with PDR [30]. Therefore, chemerin is postulated to be a crucial factor in the pathogenesis of retinopathy in diabetes. In accordance with few previous studies, we also found that plasma chemerins were elevated significantly in NPDR and PDR patients compared to NDR patients [31]. When retinopathy severity is taken into account, there was no significant difference in chemerin levels between PDR and NPDR groups, suggesting that chemerin would be expected to exert pathogenic implications for microvascular impairment at early stages of DR. The potential mechanisms for the involvement of chemerin in retinal microvascular damage are as follows: Chemrin-ChemR23 interaction may increase ROS generation and oxidative stress in vascular endothelial cells and thus impairs vascular relaxation and capillary perfusion through the activation of NADPH oxidase [32]; chemerin increases production of proinflammatory cytokines, which in turn induces leukocyte activation and migration further contributing to the retinal inflammatory and DR [24, 27]; chemerin promotes vascular endothelial permeability, proliferation, and angiogenesis, which induces neovascularization via the MAPK pathway [33, 34]. Additionally, CMKLR1 has also been implicated to play a pathophysiological role in diabetic nephropathy (DN). It has been reported that, in the kidney of diabetic animal model, the expression of CMKLR1 was gradually upregulated with the progression of renal dysfunction [35]. Knockdown of CMKLR1 ameliorated renal damage and inflammation in DN mice [36]. Recent studies showed that CMKLR1 was activated by Wnt/beta-catenin pathway which is involved in accumulation of extracellular matrix (ECM), podocyte dysfunction, and renal fibrosis in DN [37–39]. The renoprotective effect of PPAR*γ* was partly mediated by reducing the expression level of CMKLR1 in the kidney of diabetic rats [40]. However, the pathogenic role of chemerin/CMKLR1 axis in retinal microvascular function needs to be clarified by in vivo and in vitro studies.

Diabetic neuropathy is the most prevalent complication of diabetes and afflicts nearly half of diabetic patients [41–43]. In the present study, for the first time, we evaluate the relationship between chemerin and diabetic peripheral neuropathy. The results showed that there were no significant differences in plasma chemerin levels between patients with and without peripheral neuropathy. And only diabetes duration and HbA1c were independently related to neuropathy by logistic analysis. Several previous studies showed that chemerin did not have a significant relation to cardiac autonomic neuropathy in diabetes [19]. Moreover, chemerin was found to have an antinociceptive effect in an animal model of neuropathic pain [44]. One possible reason for the lack of correlation between chemerin levels and diabetic peripheral neuropathy lies in its influence on vascular function. Recently, there was evidence implicating that the impaired microvascular vasodilation was not related to the presence of neuropathy in diabetic patients [45]. In addition, limited size and employing different measures of neuropathy might affect the correlation. So the relationship between chemerin concentration and diabetic neuropathy has yet to be established.

However, it must be pointed out that there were several limitations in our study. This clinical study was cross-sectional and cross sectional data does not infer causality. Second, in recruiting the diabetic patients for this study, we could not ensure that all subgroups were similar, such as duration of diabetes, glycemic control, medicine use and coexistence of other health problems. Third, the sample size of our study is small and the study was conducted in a single urban university hospital. Because it only included patients with T2DM, our findings could not be applicable to other populations with microvascular dysfunction independent of diabetes. Fourth, the correlation between chemerin levels and some metabolic factors including BMI, SBP, TC, LDL-C hs-CRP and HbA1c is not very strong because the coefficients were relatively low. Additionally, the commercially available chemerin ELISA we used cannot distinguish between active and inactive forms of chemerin. Assessment of biologically active chemerin is better to understand the role of chemerin in metabolic disease.

In summary, here we revealed that plasma levels of chemerin are higher in diabetic patients with microangiopathic complications when compared to those without. And higher plasma chemerin level is an independent correlate of retinopathy and nephropathy, not peripheral neuropathy in diabetic patients. More detailed and comprehensive studies are expected to define chemerin's function in microvascular complication pathophysiology, and as an earlier diagnostic or therapeutic marker.

## Figures and Tables

**Table 1:** Baseline characteristics of patients with and without microvascular complications.

	All	Without complication	With complication	p
Number (*n*)	598	131	467	NS
Age (years)	54.56(12.14)	53.91 (12.21)	54.74(12.12)	NS
Sex (male/female)	383/215	90/41	293/174	NS
Duration of diabetes (years)△	8(3,13)	2(0.8,9)	10(5,15)	<0.001
Waist circumference (cm)	87.30(9.40)	86.63 (11.12)	87.37(9.23)	NS
BMI (kg/m^2^)	25.38(3.52)	24.91(3.46)	25.51(3.52)	NS
SBP (mmHg)	127.05(14.57)	125.01(13.26)	127.63(14.88)	NS
DBP (mmHg)	79.28(6.62)	78.37(7.47)	79.53(6.35)	NS
Glycated hemoglobin (%)	8.71(1.87)	8.03(1.45)	8.91(1.92)	<0.001
Triglycerides (mmol/l) △	1.65(1.17,2.57)	1.62(1.15,2.21)	1.70(1.19,2.76)	NS
Total cholesterol (mmol/l)	4.76(1.05)	4.62(1.03)	4.79(1.05)	NS
HDL cholesterol (mmol/l)	1.09(0.33)	1.07(0.30)	1.10(0.34)	NS
LDL cholesterol (mmol/l)	2.81(0.84)	2.77(0.75)	2.82(0.87)	NS
Scr (umol/l)	61.70(23.12)	55.53(13.58)	63.44(24.89)	0.001
UAE (mg/24h)△	22.4(12.4,107.1)	13.2(9,17.0)	36.8(14.4,188.76)	<0.001
hs-CRP (mg/L) △	4.70(1.8,8.60)	3.6(1.2,6.6)	5.0 (2.0,8.8)	0.021
Chemerin (ng/ml)	114.78(43.62)	97.69(40.27)	119.54(43.37)	<0.001
Medication				
Oral hypoglycemic agents	139 (23.2%)	33(25.1%)	106(22.69%)	NS
Insulin	459 (76.8%)	98(74.9%)	361(78.31%)	NS
Hypertension (%)	364(60.8%)	71(54.19%)	293(62.7%)	NS
Microvascular complications				
Nephropathy (%)	261(43.6)			
Retinopathy (%)	209(34.9)			
Neuropathy (%)	201(33.6)			
One complication (%)	322(53.8)			
Two complications (%)	86(14.4)			
Three complications (%)	59(9.9)			

Numerical variables are expressed as the mean with SD and median (interquartile range). Categorical variables were presented as number (percentage). P-value for difference between the groups was calculated from the analysis of Student's t test.

BMI, body mass index; SBP, systolic blood pressure; DBP, diastolic blood pressure; Scr, serum creatinine; UAE, urinary albumin excretion; hs-CRP, high sensitivity C-reactive protein; NS, not significant.

△Natural logarithmic transformation was used in analysis.

**Table 2:** Characteristics of all type 2 diabetic patients by chemerin tertiles.

	First tertile	Second tertile	Third tertile	
	≤80ng/ml	80-120ng/ml	≥120ng/ml	P
Number (*n*)	113	246	239	
Age (years)	54.07(11.99)	54.27(11.89)	55.08(12.48)	NS
Sex (male/female)	76/37	165/81	142/97	NS
Duration of diabetes (years)△	5(2,11)	7(3,12)∗	9 (4,15)∗#	<0.001
Waist circumference (cm)	85.15(8.80)	87.25(8.92)	88.28(10.02)	NS
BMI (kg/m^2^)	24.35(3.96)	25.31(3.09)∗	25.94(3.60)∗#	<0.001
SBP (mmHg)	126.18(13.74)	124.02(13.92)	130.59(14.90)∗#	<0.001
DBP (mmHg)	79.09(6.65)	78.96(6.75)	79.169(6.48)	NS
Glycated hemoglobin (%)	8.59(1.72)	8.61(1.85)	8.88(1.94)	NS
Triglycerides (mmol/l) △	1.70(1.24,2.59)	1.54(1.03,2.50)	1.7 (1.21,2.76)	NS
Total cholesterol (mmol/l)	4.74(1.00)	4.64(1.08)	4.88(1.07)	NS
HDL cholesterol (mmol/l)	1.12(0.33)	1.10(0.29)	1.08(0.36)	NS
LDL cholesterol (mmol/l)	2.70(0.86)	2.79(0.85)	2.89(0.78)	NS
Scr (umol/l)	54.876(17.38)	57.22(16.37)	69.55(28.50)∗#	<0.001
UAE (mg/24h)△	15.0(9.7,32.43)	17.11(11.8,41.61)	75.22(16.2,371.45)∗#	<0.001
hs-CRP (mg/L) △	3.2(1.2,7.1)	4.5(1.77,8.94)∗	5.80(2.3,10.6)∗#	<0.001
Medication				
Oral hypoglycemic agents	22(21.2%)	67(27.2%)	48(20.1%)	NS
Insulin	64(78.8%)	165(72.8%)	162(70.9%)	NS
Hypertension (%)	63(55.8%)	135(54.9%)	166(69.4%)∗#	<0.001

Numerical variables are expressed as the mean with SD and median (interquartile range). Categorical variables were presented as number (percentage). P-value for difference between the groups was calculated from the analysis of one-way ANOVA, ∗vs. First tertile group, and # vs. Second tertile.

BMI, body mass index; SBP, systolic blood pressure; DBP, diastolic blood pressure; Scr, serum creatinine; UAE, urinary albumin excretion; hs-CRP, high sensitivity C-reactive protein; NS, not significant; UAE, urinary albumin excretion.

△Natural logarithmic transformation was used in analysis.

**Table 3:** The correlation of plasma chemerin levels with other parameters.

	Pearson correlation analysis		Multiple regression analysis	
	r	P	*β*	P
Age (years)	0.05	0.26		
Sex (male/female)	0.04	0.31		
Duration of diabetes (years)△	0.18	<0.001	0.072	0.057
Waist circumference (cm)	0.06	0.21		
BMI (kg/m^2^)	0.19	<0.001	0.104	0.006
SBP (mmHg)	0.16	<0.001	0.08	0.029
DBP (mmHg)	0.07	0.10		
Glycated hemoglobin (%)	0.09	0.03	0.047	0.20
Triglycerides (mmol/l) △	0.08	0.04	-0.035	0.36
Total cholesterol (mmol/l)	0.08	0.04	-0.006	0.93
HDL cholesterol (mmol/l)	-0.03	0.42		
LDL cholesterol (mmol/l)	0.09	0.03	0.056	0.374
Scr (umol/l)	0.36	<0.001	0.188	<0.001
UAE (mg/24h)△	0.43	<0.001	0.274	<0.001
hs-CRP (mg/L) △	0.15	<0.001	0.082	0.026

BMI, body mass index; SBP, systolic blood pressure; DBP, diastolic blood pressure; UAE, urinary albumin excretion; hs-CRP, high sensitivity C-reactive protein.

△Natural logarithmic transformation was used in analysis.

**Table 4:** Clinical characteristics in type 2 diabetic patients with normoalbuminuria, microalbuminuria, and macroalbuminuria.

	Normoalbuminuria	Microalbuminuria	Macroalbuminuria	p
Number (*n*)	131	90	38	
Age (years)	53.91 (12.21)	52.75(12.72)	55.55(13.98)	NS
Sex (male/female)	90/41	57/33	30/8	NS
Duration of diabetes (years)△	2(0.8,9)	6(5,11)∗	6.5(3,13.5)∗	<0.001
Waist circumference (cm)	86.63 (11.12)	88.53(8.53)	86.7(8.12)	NS
BMI (kg/m^2^)	24.91(3.46)	25.71(3.6)	25.73(2.7)	NS
SBP (mmHg)	125.01(13.26)	126.74(12.96)	125.84(10.58)	NS
DBP (mmHg)	78.37(7.47)	79.10(6.24)	80.02(5.21)	NS
Glycated hemoglobin (%)	8.03(1.45)	8.69(1.85)∗	9.42(1.73)∗	<0.001
Triglycerides (mmol/l) △	1.62(1.15,2.21)	3.0(1.68,4.51)∗	2.00(1.05,5.32)∗	<0.001
Total cholesterol (mmol/l)	4.62(1.03)	4.88(0.98)	4.88(0.86)	NS
HDL cholesterol (mmol/l)△	1.07(0.30)	1.02(0.28)	1.05(0.24)	NS
LDL cholesterol (mmol/l)	2.77(0.75)	2.88(0.83)	3.03(0.68)	NS
Scr (umol/l)	55.53(13.58)	68.15(25.12)∗	80.84(32.89)∗#	<0.001
hs-CRP (mg/L) △	3.6(1.2,6.6)	5.4(2.1,9.05)∗	6.3(3.6,12.5)∗	0.001
Chemerin (ng/ml)	97.69(40.27)	112.93(43.40)∗	135.08(65.42)∗#	<0.001
Medication				
Oral hypoglycemic agents	33(25.1%)	31(34.4%)	9(23.7%)	NS
Insulin	98(74.9%)	59(65.6%)	29(76.3%)	NS
Hypertension (%)	71(54.19%)	57(63.3%)	26(68.42%)	NS

Numerical variables are expressed as the mean with SD and median (interquartile range). Categorical variables were presented as number (percentage). P-value for difference between the groups was calculated from the analysis of one-way ANOVA, ∗vs. Normoalbuminuria group, and # vs. Microalbuminuria group.

BMI, body mass index; SBP, systolic blood pressure; DBP, diastolic blood pressure; Scr, serum creatinine; hs-CRP, high sensitivity C-reactive protein; NS, not significant.

△Natural logarithmic transformation was used in analysis.

**Table 5:** Clinical characteristics of type 2 diabetic subjects according to the presence and severity of diabetic retinopathy.

	No DR	NPDR	PDR	p
Number (*n*)	131	68	26	
Age (years)	53.91 (12.21)	53.95(12.44)	53.61(11.80)	NS
Sex (male/female)	90/41	38/30	15/11	NS
Duration of diabetes (years)△	2(0.8,9)	7(3,13)∗	8.0(3,14.0)∗	<0.001
Waist circumference(cm)	86.63 (11.12)	87.17(11.09)	87.23(8.43)	NS
BMI (kg/m^2^)	24.91(3.46)	24.89(2.95)	26.06(4.61)	NS
SBP (mmHg)	125.01(13.26)	128.83(15.53)	121.11(9.08)	NS
DBP (mmHg)	78.37(7.47)	79.70(5.93)	78.30(4.18)	NS
Glycated hemoglobin (%)	8.03(1.45)	8.78(2.24)∗	9.24(2.46)∗	0.001
Triglycerides (mmol/l) △	1.62(1.15,2.21)	1.58(1.06,2.40)	1.77(1.14,2.86)	NS
Total cholesterol (mmol/l)	4.62(1.03)	5.07(1.07)∗	4.81(1.04)	0.017
HDL cholesterol (mmol/l)△	1.07(0.30)	1.13(0.27)	1.11(0.27)	NS
LDL cholesterol (mmol/l)	2.77(0.75)	2.96(0.86)	2.88(0.87)	NS
Serum Creatinine (umol/l)	55.53(13.58)	51.25(14.69)∗	48.19(10.10)∗	0.02
UAE (mg/24h)△	13.2(9,17.0)	13.8(10.80,19.0)	15.0(8.9,19.1)	NS
hs-CRP (mg/L) △	3.6(1.2,6.6)	6.9(2.62,11.0)	3.80(0.92,8.45)	NS
Chemerin (ng/ml)	97.69(40.27)	112.66(38.34)∗	119.95(48.62)∗	0.007
Medication				0.015
Oral hypoglycemic agents	33(25.1%)	22(32.35%)	4(15.38%)	
Insulin	98(74.9%)	46(67.65%)	22(84.62%)	
Hypertension (%)	71(54.19%)	42(61.70%)	17(65.38%)	NS

Numerical variables are expressed as the mean with SD and median (interquartile range). Categorical variables were presented as number (percentage). P-value for difference between the groups was calculated from the analysis of a one-way ANOVA and ∗vs. No DR.

BMI, body mass index; SBP, systolic blood pressure; DBP, diastolic blood pressure; Scr, serum creatinine; UAE, urinary albumin excretion; hs-CRP, high sensitivity C-reactive protein; NS, not significant.

△Natural logarithmic transformation was used in analysis.

**Table 6:** Characteristics of the subjects with and without diabetic peripheral neuropathy.

	Without DPN	With DPN	p
Number (*n*)	131	100	
Age (years)	53.91 (12.21)	55.80(11.25)	NS
Sex (male/female)	90/41	57/43	NS
Duration of diabetes (years)△	2(0.8,9)	8.0(3.25,12.0)	<0.001
Waist circumference(cm)	86.63 (11.12)	87.34(9.87)	NS
BMI (kg/m^2^)	24.91(3.46)	25.14(2.84)	NS
SBP (mmHg)	125.01(13.26)	126.00(11.45)	NS
DBP (mmHg)	78.37(7.47)	79.09(6.37)	NS
Glycated hemoglobin (%)	8.03(1.45)	8.60(1.73)	0.007
Triglycerides (mmol/l) △	1.62(1.15,2.21)	1.54(1.21,2.24)	NS
Total cholesterol (mmol/l)	4.62(1.03)	4.69(1.06)	NS
HDL cholesterol (mmol/l)△	1.07(0.30)	1.13(0.46)	NS
LDL cholesterol (mmol/l)	2.77(0.75)	2.69(0.88)	NS
Scr (umol/l)	55.53(13.58)	52.36(14.37)	NS
UAE (mg/24h)△	13.2(9,17.0)	12.6(10.35,15.6)	NS
hs-CRP (mg/L) △	3.6(1.2,6.6)	3.8(1.1,6.5)	NS
Chemerin (ng/ml)	97.69(40.27)	100.83(26.00)	NS
Medication			
Oral hypoglycemic agents	33(25.1%)	25(25.0%)	NS
Insulin	98(74.9%)	75(75.3%)	NS
Hypertension (%)	71(54.19%)	42(42.0%)	NS

Numerical variables are expressed as the mean with SD and median (interquartile range). Categorical variables were presented as number (percentage). P-value for difference between the groups was calculated from the analysis of Student's t test.

BMI, body mass index; SBP, systolic blood pressure; DBP, diastolic blood pressure; Scr, serum creatinine; UAE, urinary albumin excretion; hs-CRP, high sensitivity C-reactive protein; NS, not significant. UAE, urinary albumin excretion;

△Natural logarithmic transformation was used in analysis.

## Data Availability

The clinical data used to support the findings of this study are available from the corresponding author upon request.
